# 晚期肺癌中*ALK*活化型改变与靶向治疗的研究进展

**DOI:** 10.3779/j.issn.1009-3419.2024.102.45

**Published:** 2024-12-20

**Authors:** Aojiao WEI, Bo JIANG, Yurong HUANG, Mengyun LIU, Jing YAN, Yuanyuan ZHAO, Wenjie HE

**Affiliations:** ^1^650500 昆明，昆明医科大学; ^1^Kunming Medical University, Kunming 650500, China; ^2^650118 昆明，昆明医科大学第三附属医院老年肿瘤科; ^2^Department of Geriatric Oncology, The Third Affiliated Hospital of Kunming Medical University, Kunming 650118, China; ^3^650032 昆明，昆明医科大学第一附属医院血液科; ^3^Department of Hematology, The First Affiliated Hospital of Kunming Medical University, Kunming 650032, China; ^4^650032 昆明，昆明医科大学呼吸与危重症医学科; ^4^Department of Respiratory and Critical Care Medicine, The First Affiliated Hospital of Kunming Medical University, Kunming 650032, China

**Keywords:** 肺肿瘤, 间变性淋巴瘤激酶, 间变性淋巴瘤激酶活化型改变, 间变性淋巴瘤激酶抑制剂, 靶向治疗, Lung neoplasms, Anaplastic lymphoma kinase, Anaplastic lymphoma kinase activation patten change, Anaplastic lymphoma kinase inhibitors, Targeted therapy

## Abstract

肺癌是我国乃至全球发病率最高的癌种，也是导致癌症死亡的主要原因。存在间变性淋巴瘤激酶（anaplastic lymphoma kinase, ALK）基因改变的患者有机会接受分子靶向治疗，以ALK为靶点的药物，即ALK-酪氨酸激酶抑制剂（ALK-tyrosine kinase inhibitors, ALK-TKIs），很大程度上延长了患者的生存期。ALK基因变异类型包括点突变、扩增、融合/重排，ALK融合较其他类型更为常见。但是，各类型的基因改变在分子靶向治疗时效果有所不同，据此，本文分别介绍了ALK基因不同变异形式的相关内容，重点介绍靶向治疗的研究进展，对未来的发展方向提出探讨。

## Abstract (en)

Lung cancer is the most common cancer in China and even in the world, and it is also the main cause of cancer death. Patients with anaplastic lymphoma kinase (ALK) gene alterations have the opportunity to receive molecularly targeted therapies. The inhibitors of anaplastic lymphoma kinase, such as ALK-tyrosine kinase inhibitors (ALK-TKIs) significantly prolong the survival of patients. ALK gene variant types include point mutation, amplification, fusion/rearrangement, and ALK fusion is more common than other types. However, the effect of different types of gene changes in molecular targeted therapy is different. Therefore, this paper introduced the relevant contents of different variants of ALK gene, focused on the research progress of targeted therapy, and proposed the future development direction.

癌症是我们面临的主要公共卫生问题，在多数癌症种类中，肺癌的发病率与死亡率位居全球第一。统计数据^[1]^显示，肺癌约占癌症相关死亡人数的1/5。非小细胞肺癌（non-small cell lung cancer, NSCLC）约占所有肺癌的85%，是最常见的病理类型，主要包括腺癌、鳞状细胞癌、大细胞癌三类^[2]^。过去几年，由于新型冠状病毒感染（corona virus disease 2019, COVID-19）暴露的不利影响或疾病的异质性，部分患者未能得到及时的诊疗，就诊时已处于肺癌晚期。间变性淋巴瘤激酶（anaplastic lymphoma kinase, ALK）基因定位于2p23，编码蛋白为I型跨膜蛋白，属于受体酪氨酸激酶（receptor tyrosine kinase, RTK）超家族中的胰岛素受体家族成员，最早发现于间变性大细胞淋巴瘤中，后来在肺癌中被探索到。ALK基因可通过4条信号通路激活下游信号分子介导致癌作用，分别是PI3K-AKT、RAS-MEK-ERK、PLCγ和JAK-STAT^[3]^。核磷蛋白（nucleophosmin, NPM）是一种主要位于细胞核仁的核输出蛋白，穿梭于细胞核和细胞质之间，参与多种生物过程。根据表达水平的不同，其可作为癌基因或抑癌基因在人类致癌的过程中发挥不同作用^[4]^。1994年，Morris等^[5]^发现了ALK与NPM的融合。形成的融合体NPM-ALK具有SRC同源物2结合域，能够与自磷酸化NPM-ALK酪氨酸残基结合，激活RAS-MEK-ERK通路，从而促进细胞的生长；抑癌基因蛋白酪氨酸磷酸酶1可导致JAK-STAT通路的下调，造成细胞生长失控；PI3K可磷酸化AKT1和AKT2，通过阻断凋亡蛋白的功能达到抗凋亡的作用^[6]^。由此可见，各通路参与调节细胞增殖和凋亡的周期进程，而驱动基因ALK能够辅助癌细胞加快这些进程，达到快速增殖、侵袭的目的。

ALK基因的活化型改变包括点突变、扩增、融合/重排，在NSCLC中，ALK融合基因最为常见，点突变更多发生于靶向治疗耐药时，而扩增较前两者罕见。近年来，由于医疗技术的进步，ALK抑制剂药物的研发使得晚期NSCLC的生存率有明显的提高且具有比化疗更小的毒副作用。针对不同类型的ALK基因改变，ALK抑制剂的治疗效果参差不齐^[7]^，ALK融合的患者无论在术前或术后，甚至是无法手术的晚期都能有不同程度的获益，但由于点突变或扩增的出现，靶向治疗疗效可能大打折扣。部分点突变的激酶位点已明确，针对这些位点再次使用靶向治疗是否还能达到令人满意的效果?答案尚不明确，但我们已经在前期的研究中看到曙光。扩增也是导致耐药的其中一个原因，但靶向治疗可能收效甚微。在本文中我们概述了ALK基因不同类型的突变，就靶向治疗研究进展进行归纳综述。部分新药已在积极研发，期望在未来能够继续延长患者生存，推动实现“肺癌慢病化管理”，将靶向治疗带入一个新的阶段，同时在耐药机制方面也需要更深入的探索。

## 1 ALK融合/重排与靶向治疗研究进展

### 1.1 ALK融合/重排的概述

ALK融合/重排是指ALK发生断裂并与其他基因发生融合，其蛋白构象改变，影响自身磷酸化，从而导致肿瘤的发生。目前已在多个癌种中发现 ALK融合，在不同癌种中发生频率也有所差异，NSCLC中为3%-5%^[8]^。在NSCLC中，ALK融合较其他改变类型更为多见，因在使用靶向药物治疗后患者平均生存期长，又被称为“钻石突变”。ALK融合的伴侣不同，其对靶向治疗的敏感性不同。ALK融合伴侣可见表1^[9⇓⇓-12]^。早在2007年，Soda等^[13]^报道了ALK的融合伴侣棘皮动物微管相关蛋白样4（echinoderm microtubule-associated protein-like 4, EML4），形成的嵌合基因EML4-ALK是第2染色体内的短臂倒置的结果，与腺癌组织学相关，其占所有ALK融合突变的85%，多发生在从不吸烟或极少吸烟者以及年轻人中^[14,15]^。EML4-ALK融合存在不同的融合亚型，最常见的为EML4变体1（E13;A20）和EML4变体3a/b（E6a/b;A20），在NSCLC中的突变率分别为33%和29%^[16]^。但有研究^[17]^表明结论与上述相反，这大概与患者的群体差异有关。

**表 1:** ALK融合伴侣的差异及靶向治疗敏感性

Type of fusion partner	Incidence rate	Sensitivity to targeted drugs
EML4^[9]^	85%	Sensitive
Non-EML4	15%	
KIF5B^[10]^	Rare	Sensitive
STRN^[11]^	Rare	Possible
H1P1^[12]^	Rare	Resistant

The types of fusion partners are generally divided into EML4 and non-EML4, the latter including KIF5B, STRN, H1P1. ALK: anaplastic lymphoma kinase; EML4: echinoderm microtubule-associated protein-like 4; KIF5B: recombinant kinesin family, member 5B; STRN: striatin; H1P1: Huntington's interacting protein 1.

### 1.2 ALK抑制剂的发展

ALK抑制剂更新迭代，近15年来已有8款ALK抑制剂在国内获批上市，第一代的克唑替尼（Crizotinib），第二代的塞瑞替尼（Ceritinib）、阿来替尼（Alectinib）、布格替尼（Brigatinib）、恩沙替尼（Ensartinib）、伊鲁阿克（Iruplinalkib）、伊奉阿克（Envonalkib），以及第三代的洛拉替尼（Lorlatinib）。ALK抑制剂出现之前，治疗多倾向于化疗，克唑替尼的诞生意味着ALK抑制剂靶向精准治疗时代的开始。客观缓解率（objective response rate, ORR）是评估抗肿瘤治疗有效性的一个重要指标，指肿瘤体积缩小达到预先规定值并能维持最低时限要求的患者比例；无进展生存期（progression-free survival, PFS）是指随机分组到首次出现疾病进展或死亡的时间^[18]^。克唑替尼于2011年8月被美国食品药品监督管理局（Food and Drug Administration, FDA）获批使用。2014年PROFILE 1014研究^[19]^发现，将克唑替尼与含铂双药化疗相比，观察到显著疗效（中位PFS：10.9 vs 7.0个月，*P*<0.001；ORR：74% vs 75%，*P*<0.001）。但由于克唑替尼的治疗存在局限，于是开辟了第二代药物，且目前在治疗方面均获得显著的成效。为了对比第一代药物克唑替尼与第二代药物的疗效开展了一系列临床研究。阿来替尼于2017年11月被FDA获批为一线用药。与ALEX^[20]^研究结果一致，ALESIA研究^[21]^中数据显示，与克唑替尼相比，阿来替尼的PFS明显延长（中位PFS：41.6 vs 11.1个月），疾病进展或死亡的风险比为0.43。2024年欧洲肿瘤内科学会（European Society for Medical Oncology, ESMO）首次公布了ALESIA 7年随访数据^[22]^，阿来替尼组7年总生存期（overall survival, OS）高达56%。至今阿来替尼仍被中国临床肿瘤学会（Chinese Society of Clinical Oncology, CSCO）指南作为I级推荐药物。eXalt3研究^[23]^表明，与克唑替尼相比，恩沙替尼显著改善了PFS（25.8 vs 12.7个月）。伊鲁阿克于2024年1月被中国国家药品监督管理局（National Medical Products Administration, NMPA）获批使用。INSPIRE研究^[24]^表明，与克唑替尼相比，伊鲁阿克获益更显著（中位PFS：27.7 vs 14.6个月），疾病进展或死亡风险下降了66%。塞瑞替尼于2017年5月获FDA批准使用。2017年III期临床研究ASCEND-4研究^[25]^公布结果，塞瑞替尼组的中位PFS达到化疗组的2倍（16.6 vs 8.1个月）。2020年ALTA试验^[26]^显示布格替尼疗效优于克唑替尼（中位PFS：24 vs 11个月）。2023年J-ALTA试验^[27]^结果进一步验证了在亚洲人群中的获益。第二代药物在治疗后期仍然出现耐药，于是第三代ALK抑制剂应运而生。在CROWN研究中，将洛拉替尼与克唑替尼的疗效进行相比，于2020年报道洛拉替尼的生存获益，其可改善PFS，降低疾病进展或死亡的风险^[28]^。在2024年公布的III期CROWN研究的5年随访数据中可以看到，中位PFS已突破60个月，5年PFS率达60%，这样前所未有的数据为靶向治疗树立了信心^[29]^。伊奉阿克（TQ-B3139）是2024年6月被NMPA批准使用的新药，在2022年ESMO^[30]^报道了相比于克唑替尼，伊奉阿克显著延长了PFS（24.1 vs 11.6个月）且降低了脑转移率（1.1% vs 11.83%, *P*=0.0049）。基于以上结果，NMPA已批准布格替尼、恩沙替尼、洛拉替尼和伊鲁阿克为一线治疗用药。

### 1.3 ALK融合/重排的治疗

2024年CSCO指南指出，晚期ALK融合阳性NSCLC的一线治疗方案中，阿来替尼、布格替尼、洛拉替尼、恩沙替尼、伊鲁阿克、塞瑞替尼、克唑替尼（前三个为优先推荐药物）作为I级推荐。在晚期ALK融合阳性NSCLC的后线治疗方案中，针对具有寡进展或中枢神经系统进展的患者，将原抑制剂方案+局部治疗，或把使用一线克唑替尼更换为阿来替尼、布格替尼、洛拉替尼、恩沙替尼、伊鲁阿克、塞瑞替尼作为I级推荐。第一代抑制剂一线治疗失败的广泛进展的患者，更换为阿来替尼、布格替尼、洛拉替尼、恩沙替尼、伊鲁阿克、塞瑞替尼；一线、第二代抑制剂治疗失败的更换为洛拉替尼；ALK抑制剂治疗失败后更换为含铂双药化疗±贝伐珠单抗作为I级推荐。晚期ALK融合阳性NSCLC靶向及含铂双药失败后且体能状态（performance status, PS）评分0-2分的患者，将单药化疗作为I级推荐^[31]^。

ALK抑制剂的副作用较化疗小，抗肿瘤效果显著，使得患者的生存期延长以及生活质量提高。针对ALK融合的肺癌患者，无论在国内还是国外的治疗中，ALK抑制剂仍为首选治疗方案。随着靶向治疗的不断发展，ALK抑制剂现在不仅用于晚期肺癌患者的一线治疗与后线治疗，在肺癌术后的辅助治疗应用中发现，与既往的传统化疗相比，其可显著降低肿瘤复发及远处转移的风险，扩大潜在治愈人群的范围。值得注意的是，尽管ALK抑制剂已经取得成功，但在治疗的后期最终都会面临耐药的挑战。期待未来有更优化的靶向药物解决当前的困境。

## 2 ALK点突变与靶向治疗研究进展

ALK点突变多见于靶向治疗耐药后，大多数位于其激酶域上。不同ALK抑制剂耐药后激发突变的位点存在差异^[32]^，其中第一代ALK抑制剂克唑替尼耐药突变L1196与G1269A最为常见。第二代ALK抑制剂耐药中最常见的是G1202R。第三代ALK-TKIs洛拉替尼可克服L1196M、G1269A与G1202R等多个ALK耐药突变并穿透血脑屏障^[33]^。虽然靶向药物在治疗中取得显著成效，但是大部分都不可避免地出现耐药，具体点突变位点内容可见表2^[34⇓⇓⇓⇓⇓⇓⇓-42]^。针对点突变部分激酶域引起的耐药，第四代药物的研发正在进行中，以应对第三代ALK-TKIs耐药后无药可用的困局。

**表 2:** ALK激酶域耐药突变位点

TKIs	Resistant mutation	Sensitive mutation
Crizotinib^[34⇓-36]^	L1196M/Q, G1123S, G1128A, T1151K/M, 1151Tins, L1152R/P, C1156Y, I1171T/N/S, F1174L/V/C/I, V1180L, L1198F/P, G1202R/del, D1203N, S1206Y/R, E1210K	L1198F, E1210K
Alectinib^[34,37,38]^	1151Tins, I1171T/N/S, F1174I, L1196M/Q, V1180L, L1198F, G1202R/del	C1156Y, F1174L/C, S1206Y/R
Brigatinib^[39]^	L1198F, G1202R/del, E1210K	L1152R/P, C1156Y, I1171T/N/S, F1174L/C, L1196M/Q, S1206Y/R
Ensartinib^[40]^	G1202R/del, E1210K	G1123S, L1198F
Ceritinib^[38,41]^	G1123S, T1151K/M, 1151Tins, L1152R/P, C1156Y, F1174L/V/C, L1198F, G1202R/del, D1203N	I1171T/N/S, L1196M, S1206Y/R
Lorlatinib^[34,42]^	L1198F, G1202R+G1269A	G1123S, T1151K/M, 1151Tins, F1174L/C, L1152R/P, C1156Y, L1196M/Q, G1202R/del, S1206Y/R, E1210K

TKIs: tyrosine kinase inhibitors.

TPX-0131是一种中枢神经渗透大环分子，对ALK野生型、ALK常见突变以及各类共发突变具有较好的抑制性。临床前研究^[43]^显示，在EML4-ALK G1202R难治耐药、G1202R/L1198F和G1202R/L1196M共发突变的细胞源性异体移植的小鼠肿瘤实验中，10 mg/kg bid剂量的TPX-013表现出明确的抗肿瘤活性。遗憾的是，在I/II期临床试验FORGE-1研究（NCT04849273）^[44]^中存在风险或不利变化，该试验已宣告终止。NVL-655是一种新型具有脑渗透性的ALK抑制剂，对ALK融合及其耐药突变具有强效的抑制作用^[45]^。ALKOVE-1^[46]^是一项I/II期研究，评估NVL-655在晚期ALK阳性NSCLC和其他实体瘤患者中的安全性和有效性。在I期试验中，纳入了93例ALK融合阳性实体瘤患者（98%为NSCLC，脑转移约58%），既往治疗中，57%接受过化疗，44%服用过克唑替尼，95%服用过第二代ALK抑制剂（多为阿来替尼），83%服用过洛拉替尼。随后接受NVL-655不同剂量的治疗。截止至2024年12月，仍未达到最大耐受剂量。研究结果显示为ORR为39%（20/51），所有患者达到部分缓解，脑转移患者ORR为54%，ALK G1202R单突变或复合突变患者的ORR为71%，中位治疗持续时间为3.4个月，部分患者治疗持续时间达12个月。在安全性方面，NVL-655耐受性良好，治疗相关不良反应较轻微。大部分治疗相关不良反应的程度为1或2级，其中发生率最高的是谷丙转氨酶（alanine aminotransferase, ALT）升高（34%，任何级别）、其次是谷草转氨酶（aspartate aminotransferase, AST）升高（30%，任何级别），便秘（16%）、恶心（12%）。尽管有患者在50 mg剂量治疗时出现≥3级的转氨酶升高但胆红素正常的情况，在治疗中断2周后恢复原剂量治疗，同样能观察到不错的治疗效果（整体部分缓解，目标病灶缩小50%，持续颅内完全缓解），并且转氨酶未再升高。II期试验在150 mg剂量水平上进行并增加未接受过任何ALK抑制剂治疗的队列^[47]^。

一开始便出现ALK点突变的情况较少，更多见于针对ALK融合靶向治疗后的点突变进而导致耐药，靶向治疗受到阻碍，可致疾病进展。第四代靶向药物的出现为耐药患者带来曙光。临床前数据表明，新型ALK抑制剂具有很好的中枢渗透作用，有望打破耐药的僵局，但由于安全性原因TPX-0131的临床试验不得不终止，有些失望，但不用绝望，其他新药也大有前景。在临床研究中NVL-655未见明显的毒副作用，且有不错的ORR。即使在试验中观察到轻微不良反应，但是经历短暂停药后不良反应开始缓解甚至消失。但最佳给药剂量以及适宜人群还需在后续临床试验中观察，且试验的OS数据尚不成熟。期待能在后续的临床试验中看到振奋的结果。可见新药物的研发是一项艰辛的工程，虽然道路是崎岖坎坷的，但大方向是前进的。

## 3 ALK扩增与靶向治疗研究进展

基因扩增的定义为染色体臂受限区域的拷贝数增加。基因扩增通常导致基因过度表达和特定产物水平的提高，从而解除其信号通路的抑制作用^[48]^。若把ALK致癌通路比作一条“大路”，那ALK抑制剂便是这条路上的“障碍物”，阻断通行，ALK扩增是以“大路”拓宽的方式，增加通行的机会。肿瘤细胞会增加ALK基因的拷贝数，导致ALK蛋白水平升高，激酶活性增强。目前已在多种肿瘤中发现ALK基因扩增，如神经母细胞瘤、肺癌等。ALK扩增在神经母细胞瘤中多见，但在NSCLC中发生较少。既往在一项600多份病例筛查^[48]^中，发现ALK扩增的患病率为0.5%。2012年Salido等^[49]^发现，在170例病例中有高达10%出现扩增，并且认为扩增与性别、年龄、吸烟史或组织学类型之间没有统计学关联且ALK扩增与预后无关，这一观点与Zito Marino等^[48,50]^的研究一致，但发现与疾病分期（I、II期）存在关联（*P*=0.04）。

ALK扩增是第一代ALK抑制剂克唑替尼耐药的机制之一，说明这类ALK扩增的发生多基于ALK融合之上。由于相关文献报道较少，ALK扩增是否与ALK融合靶向治疗的敏感性有重要关联目前尚不清楚，需要进一步的研究来证实。怎样去应对或预防ALK扩增导致的耐药仍是我们有待解决的难题。

## 4 结语

提高癌症患者的生活质量也是治疗癌症的主要目的之一，对于ALK阳性的NSCLC患者，首选靶向治疗。靶向治疗能够精准锁定靶点，最小程度甚至是不伤害自身其他细胞的同时达到控制或消除肿瘤的目的。相比既往的化疗，靶向治疗不仅延长患者生存时间还能降低不良反应的发生率，充分提高了患者生活质量，明确的治疗方案逐步实现“癌症慢病化”管理，这是靶向治疗的优势所在。近年来，靶向治疗不仅局限于晚期肺癌，在术后辅助甚至是术前新辅助治疗中也有成效，可见靶向治疗已成为肿瘤时代的主旋律。

在所有活化型改变类型中，ALK融合占主导地位，在ALK融合中更多见的是EML4-ALK。ALK抑制剂层层突破难关，现已研发至第三代，靶向治疗多为口服药，患者依从性高，为无数癌症患者带来希望，尤其是第三代洛拉替尼，中位PFS达60个月，是癌症迈向治愈的重要里程碑。在临床中，多数患者在使用第二、三代ALK抑制剂皆能达到良好的抗肿瘤效果。但部分患者在治疗后期不可避免地出现耐药，病情进展不得不再次启用化疗，这部分患者的耐药可能与点突变、扩增有关，亦或者是其他我们尚未得知的耐药机制。在未来，我们可能需要集中于新靶点以及药物的探索，实现个体化精准适应性治疗。

扩增以及点突变多见于ALK融合耐药后，致病机制不够明确，以致于非融合型改变的治疗比较单一，仍有很大的探索空间。不同的药物点突变激酶域各不相同，针对某些激酶域，第四代药物已在研发中，有望改变“无药可用”的局面。ALK扩增以及点突变耐药机制是我们未来深入研究的方向，期望为治疗带来更多可能，从而进一步全方位改善所有ALK活化型改变类型患者的生存预后以及减少甚至预防靶向治疗时发生耐药。人类已向癌症发起挑战，抗击癌症过程中我们势必在未来迈入更高阶段。
